# Relationship between the Romberg test and the Wii Fit basic balance test and cognition in athletes with concussion

**Published:** 2016-04-15

**Authors:** Nicholas G. Murray, Anthony P. Salvatore, Joe Tomaka, Rebecca J. Reed-Jones

**Affiliations:** 1 School of Health and Kinesiology, Georgia Southern University, Statesboro, Georgia, United States; 2 Department of Rehabilitation Sciences, UTEP Concussion Management Clinic, College of Health Sciences, University of Texas at El Paso, El Paso, Texas, United States; 3 College of Health and Social Services, Department of Public Health Sciences, New Mexico State University, Las Cruces, New Mexico, United States; 4 Department of Applied Human Sciences, University of Prince Edward Island, Charlottetown, Canada

**Keywords:** concussion, posture, Romberg test, Wii Fit

## Abstract

**Background:**

Approximately 30% of individuals with a sport-related concussion present with postural instability. Multiple clinical balance tests exist to diagnose postural instability; yet little is known about the potential relationship between these type of postural assessments and cognition post-concussion.

**Aim:**

The purpose of the current study was to assess the relationship between the Romberg test, the Wii Fit basic balance test (WBBT), and the composite scores on the Immediate Post-Concussion Assessment and Cognitive Testing (ImPACT) test in a sample of athletes with concussions.

**Methods:**

Fifty five post-concussed athletes (40 males) completed the Romberg test (RT) (−/+), the WBBT, and ImPACT test. WBBT performance was operationalized as the number of successfully completed trials (of 5 trials of increasing difficulty) within 30 seconds. Pearson’s and point-biserial correlations examined univariate associations among the variables.

**Results:**

The RT and WBBT were not significantly related (*r* = − 0.029, *p* = 0.832). The RT weakly correlated with ImPACT impairment scores (*r* = 0.26, *p* = 0.041), whereas WBBT the number of trials did not (*r* = − 0.20, *p* = 0.155). The RT scores were significantly correlated with ImPACT Visual Processing Speed Score (*r* = 0.27, *p* = 0.036) and Reaction Time score (*r* = 0.34, *p* = 0.006). In contrast, WBBT trials were significantly correlated with the ImPACT Visual Memory Score (*r* = − 0.41, *p* = 0.003).

**Conclusions:**

These results suggest that the WBBT and RT assess unique aspects of postural control. The RT may relate directly to single sensory cognitive and motor processing, while the WBBT may relate to multi-sensory visually driven cognitive and motor processing.

**Relevance for patients:**

Clinical balance tests could point to different cognitive impairments post-concussion.

## 1. Introduction

An estimated 1.6-3.8 million concussion injuries take place in the United States per year, with over 300,000 of those being related to sports [4,8,16,20]. Of individuals suffering from sport-related concussion, 30% experience postural instability, 75.6% report dizziness as a debilitating symptom, and 90% report headaches [11,12,20]. These commonly reported symptoms indicate that a myriad of potential brain structures could be impacted post-concussion. However, it has been proposed that these injuries could impact and cross similar cognitive and motor brain pathways [6,9,10]. As such it is of great interest to investigate the potential relationship between cognition and posture post-concussion.

Simple static postural measures, such as the Romberg test (RT) and the Balance Error Scoring System (BESS), are associated with cognitive impairments in concussed athletes and cognitive declines in older adults [11,19,21]. Moreover, these methods of assessment are readily used by Athletic Trainers and recommended by the National Collegiate Athletic Association (NCAA) in determining postural impairments post-concussion [19]. However both of these assessments have a high false positive rate and only observe postural abnormalities within 72 hours post-concussion [11,22]. Recent gamification of the Nintendo Wii Fit^®^ to monitor postural differences in diseased populations has gained considerable notice [25,26]. This unique over the counter gaming system may provide a simple, objective, cost effective tool that closely resembles the functional demands of an athletic contest that stresses the three major sensory systems responsible for postural control, while simultaneously delivering a cognitive load [27]. Postural control assessments under dual-task conditions that specifically impose a cognitive task have observed postural instabilities up to 30 days post-injury [3,23,24]. These differences, whether clinically relevant or not, could be due to the inherit differences between simple tests of balance and a more comprehensive motor control task. Yet little is known about the potential relationship between these type of postural assessments and cognition post-concussion.

During the return-to-play (RTP) process the Immediate Post-Concussion Assessment and Cognitive Testing (ImPACT) test is used to reliably identify cognitive impairments in athletes with concussions [5]. The ImPACT test is a computer based neuropsychological test that measures multiple components of cognitive functioning including Verbal Memory, Visual Memory, Processing Speed, Reaction Time, and Symptom Scores [18]. Verbal and Visual Memory reflect how well one interprets or processes verbal or visual stimuli [18]. Processing Speed reflects individual ability to process information automatically without further intentional processing [18]. Reaction Time reflects simplistic sensory-motor Processing Speed [18]. Cognitive impairments identified by the ImPACT test are highly sensitive within 72 hours post-injury [5]. Moreover, the ImPACT test is widely used alongside the BESS and RT to determine impairment and monitor RTP [2,33].

Accordingly, the purpose of the current study was to assess the relationship between the Romberg Test, the Wii Fit basic balance test (WBBT), and the composite scores on the ImPACT test in a sample of athletes with concussions. Because the WBBT is an environmentally relevant postural control rehabilitation tool and it potentially requires higher order cortical sensory integration to successfully accomplish, it was hypothesized that the WBBT test would relate to the ImPACT test, while the RT would demonstrate no relationship to the ImPACT test scores.

## 2. Methods

### 2.1. Subjects

The data collected for this study were obtained from the archival records of the Concussion Management Clinic (CMC) at

The University of Texas at El Paso. A total of 156 participant records from the years 2008-2013 were evaluated for inclusion of the ImPACT test (given to all visitors) as well as the RT and WBBT. This search yielded, 55 (40 males, 15 females, average age = 19.4 years, SD = 8.7 years) individuals with complete data on all three tests. Table 1 contains participant demographics. All participants were free of any musculoskeletal and/or neuromuscular injury beyond the documented concussion injury at the time of testing as determined by self-report. In addition, no participants had a history of psychiatric illness, ADHD, or seizures as determined at the time of testing by self-report. All participants records examined self-reported no prior history of concussion within the past six months beyond the documented concussion injury at the time of testing. All participants had a confirmed concussion by a certified health professional and were referred and tested at the CMC within 24-48 hours post-injury. Participants signed an informed consent prior to all data collection and evaluation that was approved by the institute’s ethics board for the use of human subjects (Protocol No. CMC IRB 7963-13 and 285278-3).

### 2.2. Procedures

Once referred to the CMC, participants were interviewed by a trained clinician regarding their health history demographics, and events surrounding the concussion-inducing injury. During the same testing time, all participants completed the RT, ImPACT test, and the WBBT. All of the assessments were given in a randomized order. The RT evaluated balance by having individuals standing as still as possible without deviating from the standing position, regardless of foot position, while experiencing differing visual (eyes open/eyes closed) conditions [13,14,28]. Specifically, the test asks participants to place their feet together with hands close to the body and to stand quietly without moving or swaying. Trained clinicians evaluate participants for signs of deviation or sway within both visual conditions. Scores are based off the presence (+) or absence (–) of postural sway. A positive Romberg sign reflects the individuals evidencing abnormal deviations or taking a step either in the quiet standing eyes open or eyes closed, while a negative score is little to no deviations from the quiet standing position [15,35]. Prior to administration, all participants were given a brief time to practice standing in the different visual conditions.

The ImPACT test was administered to all participants randomly from the list of assessments. The procedures of the ImPACT test can be found in another publication [30]. Following administration, each participant’s scores are contrasted with normative data for each ImPACT composite score for the participant’s age and sex group. Participants are considered impaired if they had a documented and confirmed concussion by a health professional and any of their Verbal Memory, Visual Memory, Processing Speed, Reaction Time were two standard deviations outside of normative ranges.

The WBBT requires an accompanying Wii Balance Board and involves shifting weight mediolaterally to direct one’s center of pressure and to adjust set target areas on a visual display. Specifically, the game requires participants to shift weight to the left and right to maintain a red bar within a blue area denoted on the screen for three seconds [26]. The WBBT has five levels of difficulty that individuals must complete within the thirty seconds allotted for the whole test. The final score reflects how many levels the participant was able to successfully complete, along with the time it took to complete each level and a total time to complete test. All participants were given an untimed practice test to familiarize them with the game tasks.

### 2.3. Data analysis

Participant records were entered into Microsoft Excel (Microsoft, Redmond, WA, USA) and then transferred to SPSS (version 20, IBM, Armonk, New York, USA) for further analysis. Pearson’s and point-biserial correlations examined univariate associations among the primary measures; specifically the ImPACT test (i.e., overall and subcomponent composite scores), the RT, and the number of WBBT trials successfully completed. A Tukey correction for multiple comparisons was applied to the current study. Alpha level was set at 0.05 a priori.

## 3. Results

Fifty-five of 156 participants had complete data on the three primary measures. All of the participants examined participated in a sporting event and the demographics of each sport can be found in Table 1. Scores on the WBBT test tries ranged from 1 to 5 with an average of 3.84 (SD = 0.94). Only 1 participant completed the first WBBT test trial level, 2 participants completed trial level two, 17 completed trial level three, 20 completed trial level four, and 15 successfully completed trial level five (Table 2). Forty-five participants were deemed impaired via the ImPACT test, seven were considered not impaired, three were not reported (Table 3). Among those with impairment 31% showed them in Verbal Memory (17/55), 33% in Visual Memory (18/55), 31% in Visual Motor Speed (17/55), and 35% in Reaction Time (19/55) (Table 3). Furthermore, 20% suffered from a single impairment, 29% from double impairment, 11% had three impairments, 11% had four impairments, and 2% had five impairments (Table 3).

**Figure 1.**
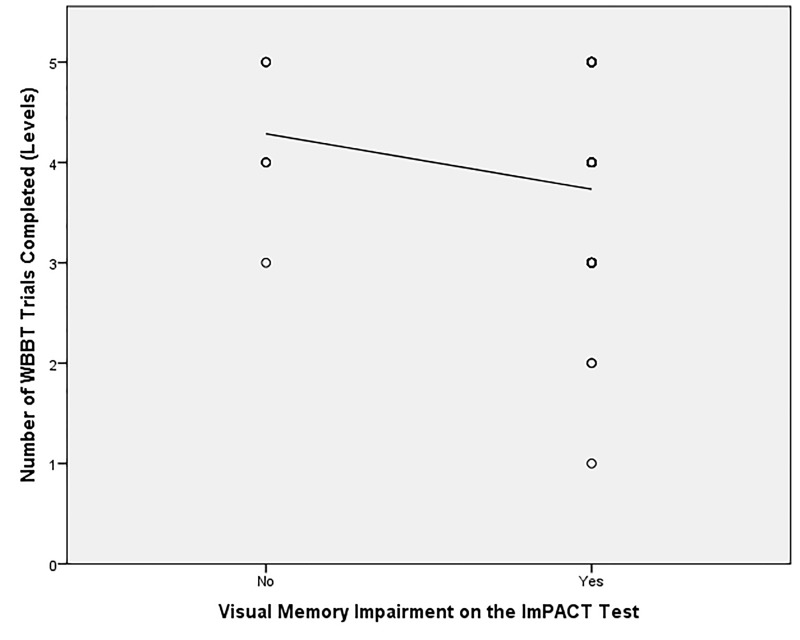
Correlation graph of the Number of WBBT trials completed and the classification of impairment on the Visual Memory on the ImPACT Test. Note the regression line added for presentation purposes to display the negative significant correlation between the variables. The circles are collapsed data points for all participants. WBBT = Wii Fit basic balance test; ImPACT = Immediate Post-Concussion Assessment and Cognitive Testing.

As Table 4 shows, the RT correlated with the ImPACT test impaired status (impaired or not impaired) (*r* = 0.261, p = 0.041). The number of WBBT test trials completed did not correlate with with the ImPACT test impaired status (*r* = –0.029, p = 0.83). An interaction was not detected between the RT and the WBBT test trials completed (*r* = 0.168, p = 0.101). Table 4 also shows correlations between the RT and ImPACT subcategory composite scores. As shown, the RT positively correlated with Visual Processing Speed (*r* = 0.266, *p* = 0.036), and Reaction Time (*r* = 0.343, *p* = 0.006). As such, the classification of impairment (0 = no impairment present, 1 = impairment present) on Visual Processing and Reaction Time increased as the classification of a positive Romberg Score (0 = negative Romberg sign; 1 = positive Romberg sign) increased. In contrast, the number of WBBT trials completed were negatively correlated with Visual Memory (*r* = *−* 0.410, *p* = 0.003). As such, as the classification of impairment (0 = No impairment present, 1 = impairment present) on Visual Memory increased, the number of WBBT trials (Level 1-5) completed decreased. Thus if a participant presented with a Visual Memory impairment they completed fewer trials on the WBBT (Figure 1).

**Table 1.** Sport type and level of participation demographics (n = 55)

Sport	# of sport
Football	23
Hockey	3
Basketball	6
Cheerleader	3
Skateboard	1
Baseball/softball	7
Skiing/snow sport	1
Track and field	2
Soccer	2
Volleyball	2
Martial arts	1
Not reported	4
Total	55
Sport level	# of sport level
Elementary	1
Middle school	8
High school	25
Semi-professional	2
College	13
Not reported	6
Total	55

Note:

# the number of that particular observation

**Table 2.** Total # of Romberg test observations (+, *−*) and the # of Wii Fit basic balance test completed (n = 55)

Romberg test observation	Frequency (%)
*+*	29 (52.7)
*−*	26 (47.3)
Total	55 (100)
Wii trials completed	Frequency (%)
1	1 (1.8)
2	2 (3.6)
3	17 (30.9)
4	20 (36.4)
5	15 (27.3)
Total	55 (100)

Note: + = a positive Romberg test sign (postural issue present), *−* = a negative Romberg test sign (postural issue absent)

**Table 3.** Impaired via ImPACT, subcategories of ImPACT composite score, and number of impairments demographics (n = 55)

	Impaired via ImPACT (%)	Verbal memory (%)	Visual memory (%)	Visual motor speed (%)	Reaction time (%)
Yes	45 (81.8)	17 (30.9)	18 (32.7)	17 (30.9)	35 (63.6)
No	7 (12.7)	35 (63.6)	34 (61.8)	35 (63.6)	17 (30.9)
Not reported	3 (5.5)	3 (5.5)	3 (5.5)	3 (5.5)	3 (5.5)
Number of impairments	One (%)	Two (%)	Three (%)	Four (%)	Five (%)
Yes	11 (20)	16 (29.1)	6 (10.9)	6 (10.9)	1 (1.8)
No	41 (74.5)	36 (65.5)	46 (83.6)	46 (83.6)	51 (92.7)
Not reported	3 (5.5)	3 (5.5)	3 (5.5)	3 (5.5)	3 (5.5)

**Table 4.** Intercorrelations of the Romberg test, Wii Fit basic balance test, the ImPACT test, and the ImPACT test composite scores (n = 55)

	Romberg test	Wii fit trials completed
Romberg test	**N/A**	– 0.029
Wii fit trials completed	– 0.029	**N/A**
Impaired via ImPACT	0.261*	– 0.200
Verbal memory	0.234	– 0.119
Visual memory	0.229	– 0.410*
Visual motor speed	0.266*	– 0.250
Reaction time	0.343*	– 0.090

Note:

* significance at the 0.05 level

## 4. Discussion

The current research examined the relationship between the RT, the WBBT, and composite scores on the ImPACT test in a sample of athletes with concussions. Specific predictions were that WBBT would relate significantly to the ImPACT test because of it potentially requires higher order cortical sensory integration to successfully accomplish, whereas the RT would not. Overall, the results did not support our hypothesis. The RT demonstrated a stronger correlation with the ImPACT test than with the WBBT. Specifically, both the RT and the WBBT related to cognitive impairment at similar levels of magnitude. However, these associations were statistically significant for the RT only, and did not reach statistical significance for the WBBT.

**Figure 2.**
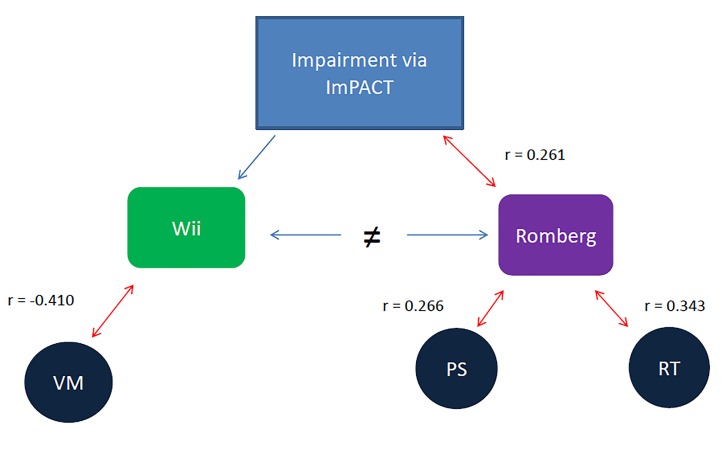
Intercorrelations of the ImPACT Test, the Wii Fit basic balance test, and the Romberg Test to each subcomponent of the ImPACT. Note: A double arrowed line indicates a significant correlation, while a single arrow line indicates no significance. VM = Visual Memory, PS = Processing Speed, RT = Reaction Time.

However, when examining each subcomponent more interesting results were the associations between the RT, the WBBT, and the ImPACT subcomponent scores. Specifically, the RT significantly related to Processing Speed and Reaction time subcomponents of the ImPACT composite score (Figure 2). Whereas the WBBT related significantly to Visual Memory subcomponent of the ImPACT test composite score (Figure 2). Overall, these results suggest that the WBBT and the RT assess unique aspects of balance and motor control. The RT relates to more simplistic reflexive lower order balance function, while the WBBT relates to higher order cortical involvement including visual stimuli identification and association as evidenced by the relationships to the respective subcomponents of the ImPACT test.

### 4.1. The Romberg test

The RT has been widely accepted as a clinical diagnostic tool for assessing neurological disorders; specifically balance dysfunction [15,17,35]. However, no data exists that has explored the relationship of the RT to a common neuropsychological assessment. The RT was significantly positively related to Processing Speed and Reaction Time impairments on the ImPACT composite scores (Table 4 and Figure 2). Prior research suggests that Reaction Time and Processing Speed are related cognitive parameters that significantly relate to psychomotor speed [7]. Psychomotor speed is the speed of which the brain can achieve simple repetitive tasks such as repetitive finger tapping [1,29]. Impairment in psychomotor speed can lead to the individual feeling slow or foggy post-concussion [1]. The relationship of Processing Speed and Reaction Time to psychomotor speed supports the notion that the RT relates to psychomotor speed. Our findings support this conclusion through the observed weak correlation between Processing Speed and the RT and the weak to medium correlation between Reaction Time and the RT.

Furthermore, psychomotor speed is modulated at the level of the brain stem and spinal cord [1]. This suggests that the RT may be a sufficient test of simple motor function that relies heavily upon somatosensory and vestibular cues for spatial orientation. In this lies one inherent limitation of the RT. The RT emulates quiet stance and cannot accurately assess environmentally relevant dynamic balance. A simple reflex based balance test such as the RT does not simulate a game-like condition. As such, suspected balance impairments may not be resolved, if measured by the RT only, and the athlete could be returning to play prematurely with lingering balance issues. Proper assessment of balance for athletes requires that they be tested using the functional demands of an athletic contest. The usefulness of visual afferent cues to avoid unwanted collision with objects or persons during an athletic contest cannot be undervalued. The absence of visual stimuli during the RT limits its ability to properly measure balance. In addition, due to the RT’s subjective grading scale it should be used with caution.

### 4.2 The Wii Fit basic balance test

The finding that the WBBT correlated negatively with Visual Memory was expected due to the coding of the raw data for the status of impairment (Figure 1). This negative relationship between the WBBT and Visual Memory suggests that as the level of difficulty increased on the WBBT a greater demand was placed upon the Visual Memory pathways. As such, the participants had an easier time completing the lower levels of the WBBT due to the hypothesized reduced cognitive demand placed upon the Visual Memory pathways. The reason for WBBT relationship to this subcomponent is likely due to the visual stimuli that the WBBT provides. It is speculated that there may be an increased cognitive load at higher levels of difficulty on the WBBT could stimulate higher order brain centers. These findings are supported by prior research that suggests appropriate cortical activity necessary to complete postural tasks relies upon on the coordination of higher order brain centers such as the thalamus and the hippocampus [31,32,34]. The Visual Memory tasks can be defined as the characteristics of our senses that pertain to visual experiences [32]. Visual Memory, requires accessing spatial orientation and location which are suggested to be associated with modality-specific, domain-specific, and feature-specific regions of Visual Perception within the thalamus and hippocampus [32]. If the WBBT does in fact tap into these brain functions then the WBBT may be an indirect measurement of Visual Perception, Visual Memory, and spatial orientation [32]. A concussive may compromise or interrupt the direct or indirect pathways leading to the thalamus and/or hippocampus. This could lead to participants having difficulty completing the higher Levels of the WBBT if they presented with a Visual Memory Impairment.

However, the lack of a significant correlation between the number of WBBT trials completed and impairment on the RT and overall impairment on the ImPACT test could tempor these claims (Table 4 and Figure 2). The lack of statistically significant between the number of WBBT trials completed and the RT could be explained by the innate application and assessment of each test. During play of the WBBT an undiscovered level of cognitive load, in conjunction with a complex postural control element, is applied and theoretically increases per level of difficulty. This could increase the demand for coordinated and sensitive postural adjustments to an even smaller visual target on the visual display. These demands may be similar to the visual and postural demands required in a sporting event where athletes engage the visual, vestibular, and somatosensory sensory systems to determine orientation in space, to intercept in-game objects, and avoid or dodge distractors. Vision is the most heavily weighted component of spatial orientation that an athlete’s body relies upon, followed by the vestibular system, and then the somatosensory system to modulate postural control during athletic competition [11]. Because the RT is assessed in a quiet upright stance position, the demands of the environmental component are minimal. In contrast, the increasing demands on the cognitive and postural systems to successfully accomplish the WBBT trial may reflect an upregulate the environmental and visual component. This is supported by the lack of relationship between the RT and the Visual Memory subcomponent of the ImPACT. As such, this our findings may indicate that the RT and WBBT are measuring two different domains of postural control. During motor movements, it is possible that certain motor patterns are repressed or overwritten given the task [36]. This could aid in explaining the lack of a relationship between the RT and the WBBT. Further research should evaluate the Center of Pressure metrics between the RT and WBBT in order to truly ascertain the underlying postural control mechanisms involved.

The lack of relationship between the WBBT and the ImPACT test could indicate two lines of analysis. First, the ImPACT test may not be ideal for measuring the higher order cognitive functions required to perform the WBBT. This explanation is possible given that the ImPACT test was designed to test identify specific areas of cognition, and not executive functioning associated with vestibular functioning. In addition, given the wide range of cognitive tests available to clinicians and neuropsychologists, certain tests not covered by the ImPACT test could relate to the higher order cognitive functioning required by the WBBT. Second, The WBBT involves the total body to achieve the desired game goals and maintain upright bipedal stance, while the ImPACT test is a seated computer based neurological assessment. Furthermore, it may be beneficial to use the WBBT in addition to the RT and ImPACT test to gain a broader perspective of potential impairment.

### 4.3. Limitations

Our study was a retrospective exploratory evaluation of patient files, along with ongoing patient evaluation. Differences in interpretation of a patient’s performance on the RT across different clinicians could have influences the reporting of a positive or negative Romberg sign. Additionally, because the data used was extracted from patient records no reliability data could be generated. No previous history of concussion was available beyond the past six months at the time of study. Moreover, the history of previous injury was obtained via self-report and due to the variable nature of self-reporting this could impact the results of the study. No baseline ImPACT scores were available at the time of the study. Lastly, the sample population was diverse and spanned multiple age ranges. Specific age categories should be investigated further.

## 5. Conclusion

The RT was significantly related to Processing Speed and Reaction Time impairments on the ImPACT composite scores. Whereas, the WBBT was significantly negatively related to Visual Memory impairments. These observed differences could be due to the divergent nature of each postural assessment. The RT may relate directly to single sensory cognitive and motor processing, while the WBBT may relate to multi-sensory visually driven cognitive and motor processing.
